# Integrated Transcriptomic and Metabolomic Analysis Reveals *VASH1* Influences Pork Quality by Regulating Skeletal Muscle Glycolysis

**DOI:** 10.3390/foods14223840

**Published:** 2025-11-10

**Authors:** Fen Wu, Yihan Fu, Jiabao Sun, Wei Zhao, Huanfa Gong, Zhe Zhang, Zhen Wang, Qishan Wang, Yuchun Pan

**Affiliations:** 1 Zhejiang Key Laboratory of Nutrition and Breeding for High-Quality Animal Products, College of Animal Sciences, Zhejiang University, Hangzhou 310058, Chinawangqishan@zju.edu.cn (Q.W.); 2 SciGene Biotechnology Co., Ltd., Hefei 230031, China; 3 Key Laboratory of Livestock and Poultry Resources Evaluation and Utilization, Ministry of Agriculture and Rural Affairs, Hangzhou 310058, China

**Keywords:** meat quality, glycolysis, transcriptomics, metabolomics, *VASH1*

## Abstract

Glycolytic potential (GP) is an important index for evaluating meat quality in the pig industry, since high muscle glycogen content generally leads to rapid postmortem glycolysis, which contributes to low meat quality. The natural differences in meat quality between Chinese local pigs (good meat quality) and Western pigs (standard meat quality) make them the ideal models for glycolysis research. Here, we investigated the mechanisms of glycolysis through comparing transcriptome and metabolome data of biceps femoris (BF) muscle between Jinhua (JH) and Landrace × Yorkshire (LY) pigs at different ages. In this research, JH pigs exhibited lower intramuscular glycogen content than LY pigs throughout the growth period (*p* < 0.05). Increased phosphorylated glycogen synthase (*p*-GS) expression indicated reduced glycogenesis capacity in JH pigs. Pathway enrichment revealed that the differentially expressed genes (DEGs) were highly enriched in glycolysis, glycogenesis, and TCA cycle pathways, but these metabolic pathways were suppressed in JH pigs. Metabolomic analysis identified increased lipids and amino acids, but carbohydrate metabolites were decreased in JH pigs. Through integrating transcriptome and metabolome data, *VASH1* was identified as a biomarker of muscle glycolysis. Mechanistically, *VASH1* knockdown promoted glucose metabolism through enhancing glycolysis and glycogenesis via the AMPK signaling pathway. Our findings provided novel insights into the genetic basis of meat quality and identify *VASH1* as a potential target for genetic selection to improve muscle glycolytic level and pork quality.

## 1. Introduction

Meat quality is a critical factor influencing consumer acceptance of pork products, determined by physicochemical properties such as pH, color, tenderness, drip loss, intramuscular fat (IMF) content, and flavor compounds [1]. However, ten decades of selective breeding for lean meat yield and growth efficiency have inadvertently compromised pork quality, leading to a higher incidence of pale, soft, and exudative (PSE) meat marked by poor water-holding capacity and pale coloration [2]. Despite its economic importance, the genetic basis of meat quality is not fully deciphered due to complex metabolic regulation.

Postmortem muscle-to-meat conversion processes and resultant meat quality variations are primarily regulated by residual glycogen reserves and glycolytic potential (GP) in skeletal muscle after slaughter [3]. Normally, the ideal ultimate pH (pH_u_) ranges from 5.5 to 5.8 [4]. Elevated muscle glycogen concentrations at slaughter had been shown to accelerate glycolysis and result in a precipitous decline in pH value, coupled with the characteristic PSE defects [5]. Although several genes including *PRKAG3* and *PHKG1* genes for the glycolysis-related traits had been reported, the genetic mechanism underlying the traits remained unclear [5,6,7]. JH pigs, a Chinese local pig breed, are renowned for superior meat quality, while LY pigs’ meat quality is normal [8,9]. These natural differences between JH and LY pigs position them as ideal comparative models for unraveling the genetic mechanisms underlying meat quality.

Due to the high resolution, quantitative accuracy and comprehensive coverage, high-throughput sequencing technologies have revolutionized livestock research field since their emergence [10,11]. Recently, transcriptome and metabolome integration analysis has emerged as a powerful framework for dissecting the complex traits by mapping dynamic interactions between genes and metabolites [12,13]. In this research, we innovatively used JH and LY pigs as models to explore the differences in muscle glycolysis. We observed that JH pigs exhibited lower intramuscular glycogen content than LY pigs at different ages. The results of metabolome and transcriptome analysis indicated that the differences between JH and LY pigs were mainly concentrated in the pathways related to the glycolysis, glycogenesis and TCA cycle pathways, which provided support for the phenotypic data. Through gene–metabolite network construction, *VASH1* was identified as a potential gene regulating glycolysis. In vitro experiments highlighted *VASH1* knockdown promoting glucose metabolism. Our study not only elucidated the molecular basis of pork quality but also offered actionable targets for precision breeding strategies in the swine industry.

## 2. Materials and Methods

### 2.1. Animal Management

Eighteen healthy JH pigs and eighteen LY pigs at 1 day of age, with randomized sex distribution, were purchased from the Jinhua Academy of Agricultural Sciences in Jinhua, China. Details including the breed, age and sex of experimental pigs are listed in Table S1. During the suckling period, the piglets stayed with their mother, and were then transferred to a pigsty with sufficient feed and water for free feeding. All experimental pigs were fed the same diet based on finishing pig requirements of the NRC (2012) in separate rooms. The formulation and chemical composition of the diet are listed in Table 1 [14]. Temperature, humidity, and light varied with the natural climate conditions. The whole experimental period lasted 180 days. At the ages of 1, 90 and 180 days, six JH and six LY pigs were slaughtered under fasting for sample collection.

### 2.2. Sample Collection

The experimental pigs were slaughtered and processed by licensed abattoir personnel using carotid artery exsanguination, in strict compliance with the Chinese national standard GB/T 17236-2019 [15]. Biceps femoris (BF) muscle, longissimus dorsi muscle (LDM) and gastrocnemius (GAS) muscle from each sample were collected and snap-frozen in liquid nitrogen, and stored at −80 °C until further. The BF samples were used for subsequent metabolomics and transcriptomics analysis simultaneously. Due to the error in the preservation process, the quality of the two 1-day-old JH pig samples failed to meet the standards and was thus unfit for omics sequencing.

### 2.3. Meat Trait Measurement

Meat quality traits were assessed in 180-day-old JH and LY pigs according to China’s agricultural industry standard NY/T 821-2019 [16]. From the left side of each carcass, LDM samples adjacent to the 12th–13th rib junction were collected for analysis, the detail procedures referred to a previous study [17]. In brief, pH values at 45 min (pH_45min_) and 24 h (pH_24h_) were measured at three locations along the same muscle sample using a calibrated pH meter (PH-STAR1, MATTHAUS, Pöttmes, Germany). Meat color was evaluated within 45–60 min using a standard colorimetric card. For drip loss determination, approximately 10 g LDM samples with a volume of 2 cm^3^ were suspended in drip-loss tubes at 4 °C, and the exudate weights were recorded after 24 h (DL_24h_) and 48 h (DL_48h_) to calculate loss rate.

### 2.4. Glycolysis Flux Measurement

The BF muscle was cut into 1 cm^3^ and fixed in 4% paraformaldehyde for over 24 h. The samples were dehydrated with gradient alcohol, then embedded and sectioned according to the periodic acid–Schiff (PAS) staining procedure (GP1039, Servicebio, Wuhan, China). Three images per tissue section were captured to visualize glycogen distribution within the muscle tissue.

To measure the glycogen and pyruvate contents, the samples were lysed by extraction solution; we then added the corresponding regent according to the manufacturer’s protocols. All experimental steps were strictly conducted in compliance with the standardized protocols provided in the product manual (BC0345 and BC2205, Solarbio, Beijing, China). Finally, the absorbance values were detected by using a Microplate Reader (Bioteck, Burlington, VT, USA).

### 2.5. Protein Extraction and Western Blot

BF muscle samples were lysed on ice in radio immunoprecipitation assay (RIPA, P0013B, Beyotime, Shanghai, China) buffer containing 1% PMSF (ST506, Beyotime) and 2% phosphatase inhibitor cocktail (P1045, Beyotime) for 30 min. The lysates were centrifuged at 12,000× *g* for 10 min at 4 °C to collect the proteins in the supernatants. The protein concentrations were measured by the BCA protein assay kit (P0009, Beyotime) and incubated at 100 °C for 10 min in 5 × SDS-PAGE loading buffer (FD002, Fdbio, Hangzhou, China). We used 20 μg of proteins per sample for electrophoresis on a 10% SDS-PAGE gel, followed by transfer to PVDF membrane (Millipore, Billerica, MA, USA). Following blocking with 5% non-fat milk for 1 h at room temperature, the membranes were sequentially incubated with specific primary antibodies at 4 °C overnight and corresponding secondary antibodies for 1 h at room temperature with three 10 min washes between each incubation step. Images were captured by the SH-Compact 523 system (SHST, Hangzhou, China) with the enhanced chemiluminescence (ECL, FD8020, Fdbio) method, and the band intensities were quantified by ImageJ software (v 1.0). GAPDH was selected as an internal control. The primary antibody information is as follows: GAPDH (ET1601-4, diluted 1:5000 Huabio, Hangzhou, China), GS (ET1611-59, diluted 1:1000, Huabio), *p*-GS (ET1602-13, diluted 1:1000, Huabio).

### 2.6. Quantitative Real-Time PCR

Total RNA of samples was extracted using TRIZOL reagent (Invitrogen, Carlsbad, CA, USA) following the manufacturer’s instructions. And 1 μg of RNA per sample was reverse-transcribed into cDNA (AE341, TransGene, Beijing, China) for quantitative real-time PCR (RT-qPCR, AQ601, TransGene) with the QIAquant96 2plex (Qiagen, Munich, Germany). The RT-qPCR primers were synthesized by Sangon Biotech (Shanghai, China) and the primer sequences are listed in Table S2. *18s RNA* and *HPRT1* were used as the internal controls in this study. Data were analyzed by using the method of 2^−ΔΔCT^.

### 2.7. Transcriptome Sequencing and Analysis

The quality of extracted RNA was determined by a 5300 Bioanalyser (Agilent, Waldbronn, Germany) and quantified using the NanoDrop 2000 (Thermo, Waltham, MA, USA). High-quality RNA (OD260/280 = 1.8~2.2, OD260/230 ≥ 2.0, RQN ≥ 6.5, 28S:18S ≥ 1.0) was used to construct sequencing libraries. Firstly, mRNA was isolated according to the polyA selection method by oligo(dT) beads and then fragmented by fragmentation buffer. Secondly, cDNA was synthesized with random hexamer primers, and then subjected to end-repair, phosphorylation and adapter addition. Libraries were constructed with target fragments of 300–400 bp cDNA by using magnetic beads. Lastly, the sequencing was performed on the NovaSeq X Plus platform at Majorbio (Shanghai, China).

The raw fastq files for RNA sequencing data are available in the NCBI SRA database under BioProject PRJNA1350905. The quality control (QC) of raw data was performed by fastp v 0.20 [18]. The filtered criterions were as follows: (i) phred score < Q20; (ii) read with adapter; (iii) read length < 15 bp; (iv) N of reads > 5. The clean data of each sample is shown in Table S1. Then, the trimmed reads were aligned to the reference genome (*Sscrofa 11.1* and *GRCm39*) using HISAT2 v 2.2.1 [19]. Later, gene expression counts were calculated with featureCounts v 2.0.3. The differentially expressed genes (DEGs) were determined using the “DESeq2” R package (v 1.46), removing sex confounding effects [20,21]. The Benjamini–Hochberg method was used for multiple comparison correction. Only genes with |log_2_(FC)| ≥ 1 and *p*adj < 0.05 were regarded as DEGs.

Gene Ontology (GO), Kyoto Encyclopedia of Genes and Genomes (KEGG) and gene set enrichment analysis (GSEA) enrichments were performed to explore the functions of DEGs. GO and KEGG enrichments were performed on the KOBAS website (http://bioinfo.org/kobas, accessed on 1 March 2025) [22]. GO terms and KEGG and GSEA pathways meeting the significance threshold *p* < 0.05 were defined as statistically enriched. Resultant data were visualized through the R package “ggplot2” (v 3.5.0).

### 2.8. Metabolomics Sequencing and Analysis

We added 50 mg samples to a 2 mL centrifuge tube with 400 μL of extraction solution (methanol/water = 4:1) containing 0.02 mg/mL of L-2-chlorophenylalanine for metabolite extraction. Samples were ground by a frozen tissue grinder under controlled conditions (−10 °C, 50 Hz, 6 min), followed by low-temperature ultrasonic extraction for 30 min (5 °C, 40 kHz) to enhance metabolite release. The samples were left at −20 °C for 30 min, then centrifuged for 15 min (4 °C, 13,000× *g*), and the resulting supernatant was transferred to the injection vial for LC-MS/MS analysis. The pooled QC samples were prepared by mixing equal volumes of all samples, then disposed and tested in the same manner as the analytic samples. It helped to represent the whole sample set, which would be injected at regular intervals (every 5–15 samples) in order to monitor the stability of the analysis.

The LC-MS/MS analysis was performed on a UHPLC-Q Exactive HF-X system (Thermo) equipped with an ACQUITY HSS T3 column (Waters). The mobile phases consisted of solvent A (0.1% formic acid in water/acetonitrile (95:5, v:v)) and solvent B (0.1% formic acid in acetonitrile/isopropanol/water (47.5:47.5:5, v:v:v)). The flow rate was 0.40 mL/min under 40 °C and the injection volume was 3 μL. The UHPLC-Q Exactive HF-X Mass Spectrometer (Thermo, Waltham, MA, USA) was used for collecting data equipped with an electrospray ionization (ESI) source operating in positive mode (3500 V) and negative mode (−3500 V). The optimal conditions were set as follows: aux gas heating temperature (425 °C), capillary temp (325 °C), sheath gas flow rate (50 psi), aux gas flow rate (13 psi), normalized collision energy (20–40–60 eV), full MS resolution (60,000), MS/MS resolution (7500). Data acquisition was performed with the Data-Dependent Acquisition (DDA) mode. The detection was carried out over a mass range of 70–1050 *m*/*z*.

The raw data were converted into the common format by Progenesis QI software (v 3.0, Waters, Milford, CT, USA) and stored in the MetaboLights database under the identifier MTBLS13217. The metabolites were identified by searching databases including HMDB (http://www.hmdb.ca/, accessed on 13 May 2025) and Metlin (https://metlin.scripps.edu/, accessed on 13 May 2025). The variables with relative standard deviation (RSD) > 30% of QC were removed for subsequent analysis. R package “ropls” v 1.6.2 was used to perform orthogonal least partial squares discriminant analysis (OPLS-DA). The metabolites with variable importance in projection (VIP) > 1 and *p* < 0.05 were considered as differentially accumulated metabolites (DAMs). Then, the KEGG pathways were enriched to explore the functions of DAMs (*p* < 0.05). All the analysis procedures were performed based on the majorbio free online cloud platform [23].

### 2.9. Weight Gene Co-Expression Network Analysis (WGCNA)

R package WGCNA v 1.72-1 was used to detect the hub genes related to specific traits [24]. Briefly, the gene expression matrices for all samples were normalized with the variance-stabilizing transformation (VST) method. Next, a scale-free network was built based on the expression matrix of genes. To keep the network consistent with scale-free topology, the soft-thresholding power value (β) was set to 11. Specially, the blockwiseModules function was used with ‘minModuleSize = 30, mergeCutHeight = 0.25’ parameters. Furthermore, the thresholds for module membership (MM) and gene significance (GS) were defined as |MM| > 0.2 and |GS| > 0.2.

### 2.10. Multi-Omics Integration Analysis

Candidate gene clusters were identified by integrating the DEGs and the hub genes. Initially, the overlapping genes were annotated using GO and KEGG enrichment. The pathways with *p* < 0.05 were considered significant. Subsequently, Pearson correlation analysis was performed between candidate genes and glycolytic-related DAMs. The absolute values of the correlation coefficient were interpreted as follows: 0.0–0.2, very weak or no correlation; 0.2–0.4, weak correlation; 0.4–0.6, moderate correlation; 0.6–0.8, strong correlation; and 0.8–1.0, very strong correlation. The gene–metabolite network was constructed using Cytoscape v3.10.0 software [25].

### 2.11. Vash1-Knockdown C2C12 Cell Construction

For exploring the function of *VASH1* in glycolysis, mouse C2C12 cells were purchased from Haoke Century Biotechnology Co., Ltd. (Hangzhou, China) and used for in vitro experiments. Cell culture procedures followed established protocols [26]. In brief, C2C12 cells were cultured in DMEM (Gibco, GrandIsland, NE, USA) supplemented with 10% fetal bovine serum (FBS, Excell) and 1% penicillin–streptomycin (Gibco) in a humidified incubator with 5% CO_2_ and 95% air at 37 °C. When the cell fusion rate was over 90%, 10% FBS was replaced with 2% horse serum to induce cell differentiation for 5 days. shRNA (5′-3′: GGGAGGACCTGATGTACAACTCGAGTTGTACATCAGGTCCTCCCTTTTTT) targeting murine *Vash1* (NM_177354.4) was synthesized by Tsingke Biotechnology Co., Ltd. (Beijing, China). For lentiviral packaging, the shRNA was cloned into the pLVX expression plasmid, co-transfected with psPAX2 and pMD2.G vectors. After that, the supernatant was collected at 24 h and 36 h, respectively. The *Vash1*-knockdown C2C12 cells were achieved by lentivirus transduction in the presence of 2 μg/mL polybrene.

### 2.12. Statistical Analysis

For assessing differences between the two groups, including the comparison of meat quality parameters, glycogen contents, and gene and protein expression levels, a two-tailed *t*-test was performed with GraphPad Prism v9.5. The data are presented as mean ± SD (standard deviation). The assumptions of normality for each group and homogeneity of variances were verified for all *t*-tests using the Shapiro–Wilk and Levene tests, respectively. Values of *p* < 0.05 were considered to indicate statistically significant differences. Significance levels were denoted as follows: *p* ≥ 0.05, no label; 0.01 < *p* < 0.05, labeled “*”; *p* < 0.01, labeled “**”. Results from representative experiments, such as micrographs, were obtained from at least three independent fields of view with similar results.

## 3. Results

### 3.1. Meat Quality and Glycogen Differential Analysis

**Figure 1:**
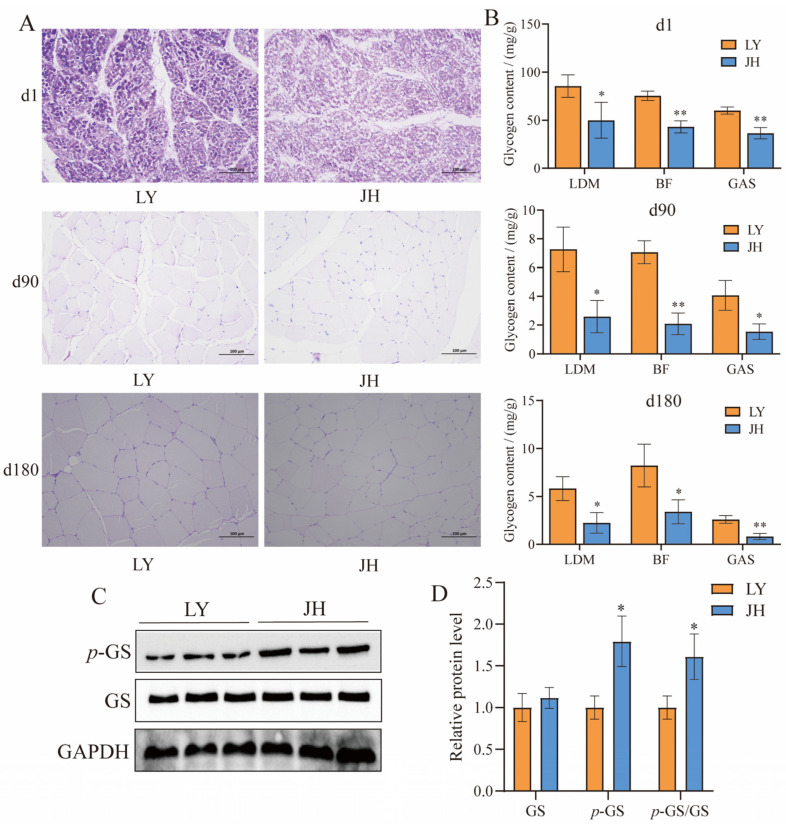
Glycogen measurements. (**A**) PAS staining of BF muscles of JH and LY pigs at d1, d90, and d180. (**B**) Statistical results of glycogen contents in BF, LDM and GAS muscles between JH pigs (*n* = 3) and LY pigs (*n* = 3). (**C**) Western blot of glycogen synthesis protein including GS and *p*-GS, *n* = 3. (**D**) Statistical results of protein expression. For assessing differences between the two groups, a two-tailed *t*-test was performed. *: *p* < 0.05, **: *p* < 0.01. Error bars represent the SD of the mean.

JH and LY pigs were sacrificed for meat quality detection at 180 days of age. As shown in Table 2, the meat color was significantly stronger in JH pigs than LY pigs (*p* < 0.05). Although without significant difference, the pH_45min_ values were slightly higher in JH pigs, while the DL_24h_ and DL_48h_ were slighter lower in JH pigs (*p* > 0.05, Table 2). PAS staining revealed significant differences in muscle glycogen content between JH and LY pigs at different ages (Figure 1A). The glycogen contents in the LDM, BF, and GAS muscles of JH pigs were lower than those in LY pigs at 1, 90, and 180 days of age at significant or extremely significant levels (*p* < 0.05 and *p* < 0.01, Figure 1B). Western blot showed the significantly elevated expression levels of phosphorylated glycogen synthase (*p*-GS) in JH pigs compared to LY pigs (*p* < 0.05, Figure 1C,D).

### 3.2. Transcriptional Specificity Analysis

A total of 34 BF muscle libraries were constructed, and the RNA sequencing results are shown in Table S1. Raw sequencing data generated an average of 56.83 million reads per library (range: 43.70–68.52 million). After QC, more than 97% of reads were obtained as high-quality clean data. Subsequently, the clean reads were aligned to the reference genome and achieved >95% mapping efficiency across all samples. The clustering heatmap demonstrated strong reproducibility between biological replicates, suggesting the reliability of our transcriptomic profiling (Figure S1A).

**Figure 2:**
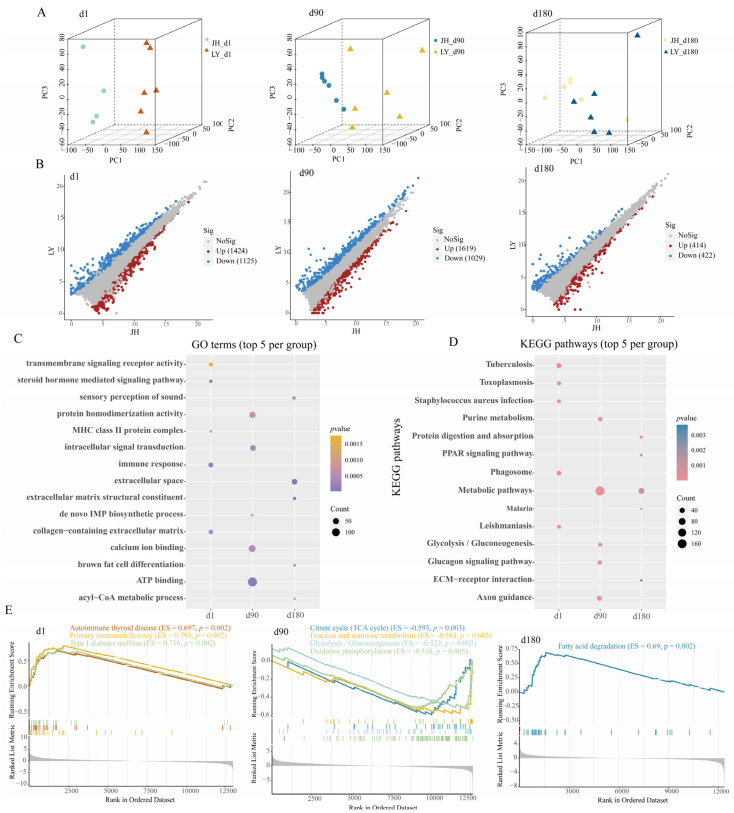
Transcriptional analysis between JH and LY pigs in three different age groups. (**A**) PCA plots between JH and LY pigs in different age groups, from left to right, are d1, d90 and d180 groups respectively. (**B**) The volcano plots of gene expression levels between JH and LY pigs in different age groups, from left to right, are d1, d90 and d180 groups respectively. Red dots signify up-DEGs, blue dots signify down-DEGs, and grey dots signify non-significance. (**C**) The dot plot shows the respective top 5 GO terms in different age groups. (**D**) The dot plot shows the respective top 5 KEGG pathways in different age groups. The gradient color represents the size of *p* value, and the size of the dot represents the size of the gene number. (**E**) The GESA visual of enriched KEGG pathways, from left to right, are d1, d90 and d180 groups respectively. ES represents enrichment score.

PCA revealed that while partial overlap existed among clusters, all biological replicates remained within the 95% confidence ellipses, with discernible inter-group separation patterns (Figure S1B). The clades observed in the NJ tree were consistent with the PCA results (Figure S1C). As shown in Figure 2A, JH pigs and LY pigs were separated significantly on the first three principal components in each age group. After differential expression analysis, there were 2549 (1424 up-regulated and 1125 down-regulated), 2644 (1615 up-regulated and 1029 down-regulated), and 836 (414 up-regulated and 422 down-regulated) DEGs in the d1, d90, and d180 groups respectively (Figure 2B, Tables S3–S5). The qPCR results were found to be consistent with the expression trends observed in RNA-seq (Figure S2). Chromosomal mapping showed an even distribution of DEGs across all autosomal regions (Figure S3).

For exploring the functions of DEGs, we performed GO and KEGG enrichment analysis. The significantly enriched GO terms and KEGG pathways by DEGs in different age groups are listed in Tables S6–S8. In the d1 group, the DEGs were predominantly mapped to immune-related terms and pathways, including immune response (GO: 0006955), tuberculosis (ssc05152), staphylococcus aureus infection (ssc05150), toxoplasmosis (ssc05145), and leishmaniasis (ssc05140) (Figure 2C,D, Table S6). In the d90 group, DEGs showed pronounced enrichment in glucose metabolism-related terms and pathways, including ATP bonding (GO: 0005524), the glucagon signaling pathway (ssc04922), and glycolysis/gluconeogenesis (ssc00010) (Figure 2C,D, Table S7). In the d180 group, DEGs were converged on lipid biosynthetic processes-related terms and pathways, including brown fat cell differentiation (GO: 0050873) and the PPAR signaling pathway (ssc03320) (Figure 2C,D, Table S8). These results illustrated breed-specific transcriptional trajectories during development. Compared with LY pigs, disease- and immune-related KEGG pathways (including autoimmune thyroid disease, primary immunodeficiency and type I diabetes mellitus) were activated at d1, glycometabolism-related KEGG pathways (including TCA cycle, fructose and mannose metabolism, glycolysis/gluconeogenesis and oxidative phosphorylation pathways) were suppressed at d90, and a fat deposition-related KEGG pathway (fatty acid degradation) was activated at d180 in JH pigs (Figure 2E).

### 3.3. Hub Gene Selection in JH Pigs

Based on the standardized gene expression matrix, we performed WGCNA to identify functionally related gene modules. No outlier samples were found in the hierarchical clustering of samples, ensuring data reliability for subsequent analyses (Figure S4A). A soft threshold of 11 was used to assure a scale-free network distribution of the gene expression matrix (Figure S4B). Through dynamic tree cutting and module merging, we identified 14 distinct co-expression modules (Figure S4C). Module size varied substantially, with the brown module containing the largest gene set (*n* = 7918), whereas the dark turquoise module represented the smallest cluster (*n* = 97) (Figure S4D). Correlation analysis showed the module–sample specificity, suggesting that the modules had sample preference (Figure S4E).

**Figure 3:**
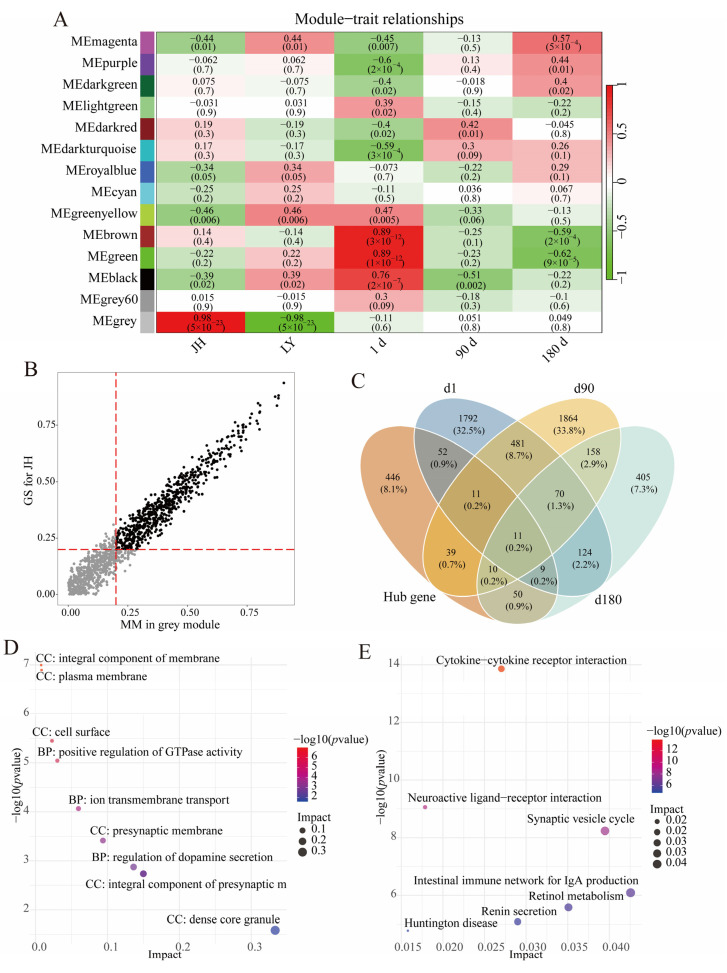
Co-expression module construction and hub gene selection. (**A**) Heatmap showing the relationship between module and phenotype. Red represents positive correlation; green represents negative correlation. (**B**) Scatterplot of GS for pig population vs. MM in grey module. The *x*-axis represents MM in grey module, and the *y*-axis represents GS for JH pigs, the red lines represent the thresholds of |MM| > 0.2 and |GS| > 0.2, respectively. (**C**) The Venn diagram shows the hub DEGs in JH pigs. (**D**) The dot plot shows the GO terms enriched by hub DEGs. (**E**) The dot plot shows the KEGG pathways enriched by hub DEGs.

Phenotypic metadata encompassed breed and age for each sample. Module–phenotype associations were assessed by calculating Pearson correlation coefficients between module eigengenes and phenotypic variables. Notably, the grey module exhibited the strongest positive correlation with JH pigs (r = 0.98, *p* = 5 × 10^−23^, Figure 3A). Hub genes are a series of genes with the highest degree of connectivity in a module and determine the characteristics of the module to a certain extent, which are identified using thresholds of |MM| > 0.2 and |GS| > 0.2. In the grey module, 627 genes that met the threshold criteria were identified as hub genes (Figure 3B, Table S9). The intersection of these hub genes and DEGs in three different age groups yielded 181 overlapping hub DEGs, with subgroup-specific counts of 82 (d1 group), 71 (d90 group), and 79 (d180 group) hub DEGs (Figure 3C, Table S9). The functional analysis of these 181 hub DEGs showed significant enrichment in signal conduction-related GO terms and KEGG pathways, including integral component of presynaptic membrane (GO: 0099056), positive regulation of GTPase activity (GO: 0043547), ion transmembrane transport (GO: 0034220), cytokine–cytokine receptor interaction (ssc04060), neuroactive ligand–receptor interaction (ssc04080), and synaptic vesicle cycle (ssc04721) (Figure 3D,E, Table S10).

### 3.4. Metabolic Diversity and Composition Analysis

**Figure 4:**
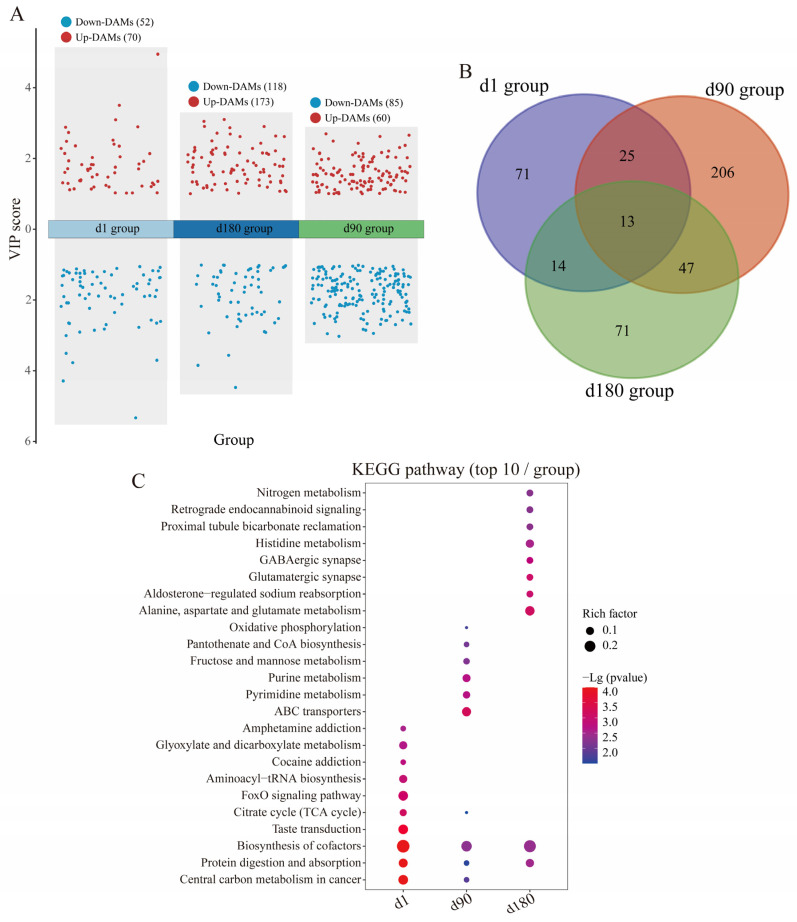
Metabolomic analysis between JH and LY pigs in three different age groups. (**A**) The volcano plots of DAMs between JH and LY pigs in d1, d90 and d180 groups, respectively. The red dots signify up-DAMs and blue dots signify non-significance. (**B**) The Venn analysis of DAMs across d1, d90 and d180 groups. (**C**) The dot plots show the KEGG pathways enriched by DAMs between JH and LY pigs in d1, d90 and d180 groups, respectively. The gradient color represents the size of the *p* value, and the size of the dot represents the size of the metabolite number.

PCA and OPLS-DA robustly differentiated JH and LY pigs at different ages, with abundant metabolites driving distinct clustering patterns consistently observed in both positive and negative ion modes (Figure S5). There were 52, 118 and 85 up-regulated metabolites and 70, 173 and 60 down-regulated metabolites between JH and LY pigs in d1, d90 and d180 groups, respectively (Figure 4A, Tables S11–S13). According to the DAM classification, there was a relatively higher ratio of amino acids in the d1 group (Figure S6A), phospholipids and carboxylic acids in the d90 group (Figure S6B), and phospholipids in the d180 group (Figure S6C). Among these, the Venn diagrams showed there were 13 DAMs shared among these three groups, including isovaleric acid and betaine (Figure 4B).

Pathways enrichment analysis identified key metabolic pathways across different age groups. It is worth noting that glycolysis-related pathways were significantly enriched in each age group, including citrate cycle (TCA cycle, map00020), FoxO signaling pathway (map04068), cAMP signaling pathway (map04024), glucagon signaling pathway (map04922), PI3K-Akt signaling pathway (map04151), mTOR signaling pathway (map04150), fructose and mannose metabolism (map00051), and oxidative phosphorylation (map00190) (Figure 4C, Tables S14–S16). These findings were consistent with the transcriptome analysis.

### 3.5. Key Candidate Gene Screening

**Figure 5:**
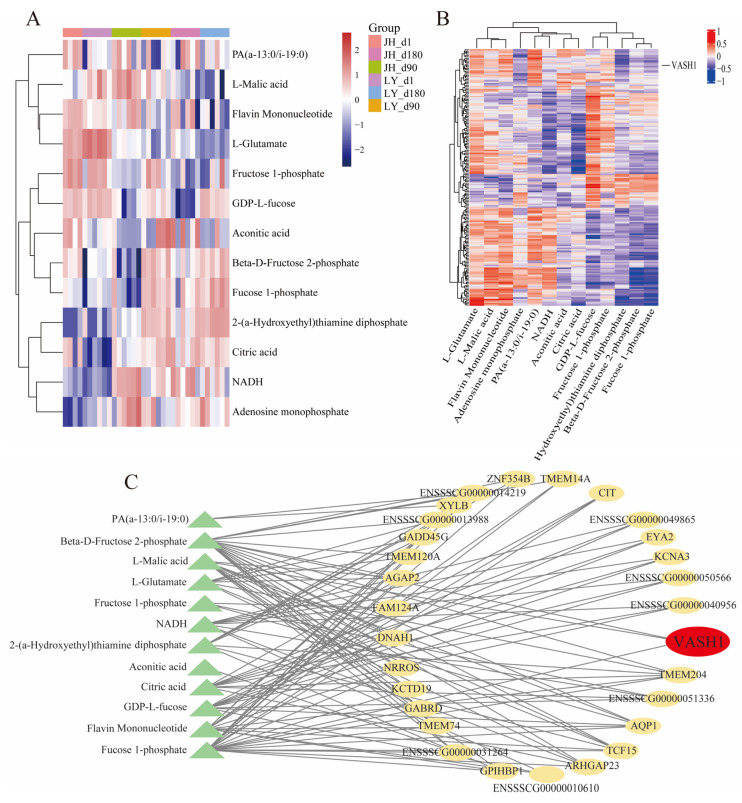
Identification of potential genes that influenced glycolysis. (**A**) Heatmap of the accumulation of 13 glycolysis-related metabolites. (**B**) The heatmap of Pearson correlation between 181 hub DEGs and glycolysis-related DAMs. (**C**) The interaction network between 29 potential genes and glycolysis-related metabolites.

In order to identify the specific regulatory gene of skeletal muscle glucose metabolism, a Pearson correlation test was used to analyze the connection between 181 hub DEGs and glycolysis-related DAMs. Only DAMs significantly enriched in glycolysis-related pathways were selected for coefficient analysis. The abundance profiles of these glycolysis-related DAMs between JH and LY pigs are visualized in Figure 5A. Interestingly, JH pigs exhibited lower glycolytic metabolite abundances, including citric acid and beta-D-fructose 2-phosphate, suggesting potential divergence in skeletal muscle energy metabolism. Correlation analysis identified 29 hub DEGs that showed moderate-to-strong associations (|r| ≥ 0.4, *p* < 0.05) with glycolysis-related metabolites (*n* ≥ 3) (Figure 5B, Table S17). And a comprehensive interaction network integrating 29 candidate genes and 13 glycolysis-related DAMs was further reconstructed (Figure 5C). After excluding low-abundance DEGs (defined as transcripts with average expression < 500 baseMean, Figure S6A–C), we prioritized *VASH1* for in vitro experiments based on the prior literature reports.

### 3.6. Vash1 Knockdown Promotes Glycolysis and Glycogenesis Within C2C12 Cells

**Figure 6:**
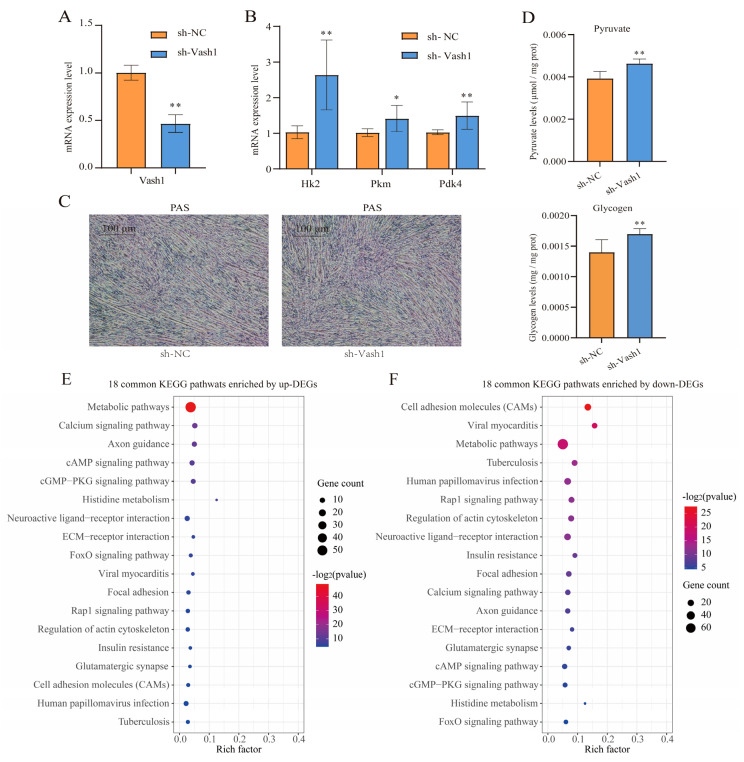
*Vash1* knockdown promoted glycolysis and glycogenesis in C2C12 cells. (**A**) The efficiency of *Vash1*-knockdown shRNA, *n* = 3. (**B**) *Vash1*-knockdown shRNA promoted glycolysis-related genes’ (*Hk2*, *Pkm* and *Pdk4*) expression, *n* = 3. (**C**) PAS staining of glycogen in *Vash1*-knockdown myotubes and statistical result, *n* = 3. (**D**) The statistical result of pyruvate level within *Vash1*-knockdown myotubes, *n* = 3. (**E**) Eighteen common KEGG pathways enriched by up-DEGs. (**F**) Eighteen common KEGG pathways enriched by down-DEGs. For assessing differences between the two groups, a two-tailed *t*-test was performed. *: *p* < 0.05, **: *p* < 0.01. Error bars represent the SD of the mean.

Multiple sequence alignment showed a high conservation of *Vash1* among porcine, human, and murine orthologs, providing justification for functional validation in C2C12 myoblasts (Figure S7). As shown in Figure 6A, *Vash1*-targeting shRNA (sh-*Vash1*) achieved a significant suppression of *Vash1* expression with over 50% knockdown efficiency. *Vash1* knockdown increased expression of *Hk2*, *Pkm*, and *Pdk4* mRNA versus sh-NC controls (Figure 6B). In addition, *Vash1* knockdown increased glycogen deposition (Figure 6C) and pyruvate levels (Figure 6D). These coordinated metabolic shifts suggested *Vash1* as a key suppressor of glycolytic processes and glycogenesis in myotubes.

We performed RNA sequencing on sh-NC and sh-*VASH1* cells to investigate the molecular mechanisms of *VASH1*-mediated biological processes. The sequencing results are shown in Table S1. The PCA plot revealed a distinct separation between the sh-NC and sh-*VASH1* groups, and the PC1 and PC2 accounted for 95% and 2% of the total variance, respectively (Figure S8A). The NJ tree separated all samples into two distinct branches (Figure S8B). Compared to the sh-NC group, there were 479 up-regulated and 1249 down-regulated genes in the sh-*VASH1* group (Figure S8C, Table S18). Functional enrichments revealed that there were 18 KEGG pathways co-enriched by up-DEGs and down-DEGs significantly, including the insulin resistance (mmu04931) pathway and FoxO signaling pathway (mmu04068) (Figure 6E,F, Tables S19 and S20). Meanwhile, the glycolysis/gluconeogenesis (mmu00010) pathway and AMPK signaling pathway (mmu04152) were significantly enriched by up-DEGs (Table S19). The type I diabetes mellitus (mmu04940) pathway and PI3K-Akt signaling pathway (mmu04151) were significantly enriched by down-DEGs (Table S20). Furthermore, the citrate cycle (TCA cycle, mmu00020) pathway and pyruvate metabolism (mmu00620) were enriched by up-DEGs, though this association approached but did not reach statistical significance (*p* = 0.051 and *p* = 0.069, Table S19). These findings highlight the critical role of *VASH1* in regulating glucose metabolism within skeletal muscle.

## 4. Discussion

Previous studies had established that skeletal muscle glycolytic potential highly related to the ultimate meat quality, with elevated glycogen levels resulting in low meat quality [5,6]. After slaughter, the organism begins anaerobic respiration, which breaks down stored glycogen into lactate and leads to a progressive decrease in pH values. The pH decline triggers the denaturation of sarcoplasmic and myofibrillar proteins, thereby ultimately compromising meat color, palatability, and water-holding capacity [27,28]. In this research, comparative analysis revealed that JH pigs exhibited lower muscle glycogen levels compared to LY pigs at different ages, which may partially explain the reason for pH_45min_, pH_24h_, DL_24h_, DL_48h_ and meat color being relatively superior in JH pigs. The pH values decreased over time and stabilized around 24 h post-mortem due to glycogen depletion [29]. However, no significant difference in pH and drip loss values was observed between JH and LY pigs, which might be due to the limited sample size. This natural difference in glycogen content and meat quality made the JH and LY pigs the ideal models for exploring the genetic mechanisms underlying glycolysis and meat quality.

Transcriptomic analysis revealed that glycolysis, gluconeogenesis, TCA cycle and oxidative phosphorylation were suppressed in JH pigs compared to LY pigs, which explained the reason why JH pigs had lower glycogen contents in their skeletal muscle. According to WGCNA results, hub genes were significantly enriched in signal conduction functions such as GTPase activity and ion transmembrane transport. It is known that the molecular signaling involved in inducing glucose transported into muscle is complex and involves a variety of signaling molecules, including Ca^2+^ and NOS in the proximal part of the signaling cascade, as well as GTPases, Rab, and cytoskeletal components in the distal part [30,31]. Multiple signaling cascades, including MAPK, PI3K-Akt and Wnt pathways, had been implicated in the regulation of glucose metabolism in skeletal muscle [32,33,34]. These findings suggested that the difference in the activation of signaling conduction might be the reason for the divergence in glucose metabolism within skeletal muscle between JH and LY pigs.

Metabolomic profiling revealed significant disparities across carbohydrates, amino acids, nucleotides and lipids between JH and LY pigs. These compositional variations contributed to flavor compounds through biochemical transformations such as Maillard reaction cascades and lipid oxidation pathways [35]. The composition and content of fatty acids and amino acids in muscle affected the pork flavor and nutritional value directly [29,36]. Phospholipids were more prone to flavor generation during lipid oxidation than triglyceride [37]. Creatine was an important energy metabolite and could improve pH value and water-holding capacity and reduce shear force by delaying glycolysis [38]. In our results, the contents of flavor amino acids, phospholipids and creatine contents in JH pigs were significantly higher than those in LY pigs, providing biochemical substantiation for their superior organoleptic qualities.

In skeletal muscle physiology, glucose primarily originates from the systemic circulation and undergoes GLUT4-mediated translocation into myocytes [30]. The metabolic fate of intramuscular glucose is primarily partitioned into two distinct pathways: (1) glycogenesis for energy storage via glycogen synthase activation, and (2) glycolysis for energy generation [39,40]. Under aerobic conditions, glucose is generally assumed to be burned fully by tissues via the TCA cycle to carbon dioxide; alternatively, glucose can be catabolized anaerobically via glycolysis to lactate [41]. In this research, glycolysis, gluconeogenesis, the TCA cycle and oxidative phosphorylation were suppressed in JH pigs. In addition, especially in the d90 group, the abundance of glycolytic metabolites such as citric acid and aconitic acid in JH pigs was significantly lower than in LY pigs. Our results showed that JH pigs exhibited significantly attenuated glycolytic flux capacity relative to LY pigs, which mechanistically accounted for their superior meat quality.

*VASH1*, a negative feedback regulator of angiogenesis, was reported to play roles in regulating glucose tolerance and insulin resistance in recent studies [42,43]. A paper reported that knockout of *VASH1* in mice resulted in reduced expression of insulin receptor in white adipose tissue [44]. It is known that the suppression of the insulin signaling pathway could inhibit the PI3K-Akt signaling pathway, down-regulate GLUT4 expression, and decrease the glucose transport ratio [45,46]. In our results, *VASH1* was highly associated with glycogen synthesis and glycolysis, and *VASH1* knockdown suppressed the PI3K-Akt signaling pathway in cells. Thus, we considered that *VASH1* knockdown could influence the glucose transport ratio into skeletal muscle.

AMPK is a sensor of cellular energy status that is activated by energy stress, signaled by rising AMP and ADP coupled with falling ATP, which adjusts metabolism to restore energy homeostasis [47]. Previous studies had identified that the enhancement of the AMPK signaling pathway was related to accelerated skeletal muscle glycolysis [48,49]. Our results reflected that *VASH1* knockdown activated the AMPK signaling pathway and glycolysis/gluconeogenesis pathway, increasing mRNA expression of *HK2*, *PKM* and *PDK4* and promoting glycogen synthesis. Despite reduced glucose uptake efficiency, we recognized that the activation of the compensatory mechanism triggered up-regulation of the AMPK signaling pathway, thereby augmenting glycolytic flux to sustain cellular energy homeostasis. A growing body of evidence reported that glucose transport efficiency operated independently from glycolytic activity [50,51]. The activation of multi-tiered emergency response systems comprising alternative energy pathways serves as a critical manifestation of metabolic flexibility that sustains glucose metabolic homeostasis in skeletal muscle. However, the details of emergency response systems comprising alternative energy pathways induced by *VASH1* are another crucial topic to address. To further validate the function of *VASH1* in muscle glycolysis, in vivo experiments should be conducted in future studies.

## 5. Conclusions

In conclusion, we observed reduced intramuscular glycogen content and diminished glycogen synthesis in JH pigs compared to LY pigs at different ages. Transcriptomic analysis revealed DEGs primarily associated with glucose metabolism including glycolysis, glycogenesis and TCA cycle pathways. Metabolomic profiling uncovered DAMs mainly involved in the TCA cycle, glucagon signaling pathway, fructose and mannose metabolism and oxidative phosphorylation pathways. Integrated multi-omics analysis and cellular experiments suggested that *VASH1* knockdown promotes glucose metabolism via the AMPK signaling pathway. This research provided a mechanistic foundation for precision improvement strategies in pig meat quality.

## Figures and Tables

**Table 1:** The formulation and chemical composition of the pig diet.

Items	Values
Formulation	
Maize	47.6%
Soybean meal	8.5%
Alfalfa, dehydrated	1.5%
Wheat, soft	8.0%
Barley	6.0%
Wheat feed flour	4.0%
Rice bran, oil > 5%	16.0%
Rice, polished, broken	5.0%
Limestone	0.9%
Premix	2.5%
Chemical composition	
Dry matter (DM)	87.3%
Crude protein (CP)	16.2%
Crude fiber (CF)	4.3%
Ether extract (EE)	5.7%
Ash	5.0%
Neutral detergent fiber (NDF)	13.0%
Acid detergent Fiber (ADF)	4.7%
Total calcium (Ca)	0.7%
Total phosphorus (P)	0.8%
Digestible energy (DE)	3358.2 Kcal/kg
Metabolic energy (ME)	3226.1 Kcal/kg
Gross energy (GE)	3892.0 Kcal/kg

**Table 2:** Comparative analysis of meat quality between JH and LY pigs.

Traits	JH (*n* = 6)	LY (*n* = 6)	*p*
pH_45min_	6.66 ± 0.12	6.54 ± 0.62	0.06
pH_24h_	5.78 ± 0.25	5.71 ± 0.18	0.60
DL_24h_	3.85 ± 0.01	4.38 ± 0.01	0.34
DL_48h_	6.76 ± 0.01	6.90 ± 0.01	0.81
Meat color	3.00 ± 0.65 ^a^	2.04 ± 0.37 ^b^	0.01

^a,b^ Data with different superscript letters are significantly different (*p* < 0.05). Note: Data are expressed as the means ± SD.

## Data Availability

The original contributions presented in the study are included in the article/Supplementary Materials. Further inquiries can be directed to the corresponding author.

## Supplementary Materials

The following supporting information can be downloaded at https://www.mdpi.com/article/10.3390/foods14223840/s1, Figure S1. The cluster analysis of all samples, including JH and LY pigs at three different age stages (d1, d90 and d180). Figure S2. The expression level of DEGs between JH and LY pigs. Figure S3. The distribution of DEGs across chromosomes. Figure S4. WGCNA of transcriptome. Figure S5. The cluster analysis of all samples. Figure S6. The classification of metabolites. Figure S7. The homologous identity analysis of VASH1 gene among pig, human and mouse. Figure S8. Transcriptomic analysis of Vash1-knockdown cells. Table S1. The quality assessment of RNA sequencing data. Table S2. qPCR primer sequence. Table S3. The DEGs between JH and LY pigs in d1 group. Table S4. The DEGs between JH and LY pigs in d90 group. Table S5. The DEGs between JH and LY pigs in d180 group. Table S6. The GO terms and KEGG pathways enriched by DEGs in d1 group. Table S7. The GO terms and KEGG pathways enriched by DEGs in d90 group. Table S8. The GO terms and KEGG pathways enriched by DEGs in d180 group. Table S9. The hub DEGs in the grey module. Table S10. The GO terms and KEGG pathways enriched by 181 hub DEGs. Table S11. The DAMs between JH and LY pigs in d1 group. Table S12. The DAMs between JH and LY pigs in d90 group. Table S13. The DAMs between JH and LY pigs in d180 group. Table S14. The KEGG pathways enriched by DAMs between JH and LY pigs in d1 group. Table S15. The KEGG pathways enriched by DAMs between JH and LY pigs in d90 group. Table S16. The KEGG pathways enriched by DAMs between JH and LY pigs in d180 group. Table S17. The Pearson correletion between DAMs and hub DEGs. Table S18. The DEGs between sh-Con vs sh-Vash1 groups. Table S19. The KEGG pathways enriched by up-DEGs in Vash1-knockdown group. Table S20. The KEGG pathways enriched by down-DEGs in Vash1-knockdown group.
