# Supplementary material for: Integrated Transcriptomic and Metabolomic Analysis Reveals VASH1 Influences Pork Quality by Regulating Skeletal Muscle Glycolysis
Source: Foods. 2025 Nov 10;14(22):3840. doi: 10.3390/foods14223840 (PMC12651661; doi:10.3390/foods14223840)
Supplement: Supplementary file 1 [file foods-14-03840-s001.zip › Supplementary Figure.pdf]

Integrated transcriptomic and metabolomic reveals *VASH1* influences pork quality by regulating skeletal muscle glycolysis

Fen Wu[a], Yihan Fu[a], Jiabao Sun[a], Wei Zhao[a,b], Huanfa Gong[a], Zhe Zhang[a], Zhen Wang[a], Qishan Wang[a,c,*], Yuchun Pan[a,c,*]

[a]Zhejiang Key Laboratory of nutrition and breeding for high-quality animal products, College of Animal Sciences, Zhejiang University, Hangzhou 310058, China

[b]SciGene Biotechnology Co., Ltd., Hefei 230031, China

[c]Key Laboratory of Livestock and Poultry Resources Evaluation and Utilization, Ministry of Agriculture and Rural Affairs, Hangzhou 310058, China

* Corresponding author: Qishan Wang: wangqishan@zju.edu.cn

Yuchun Pan: panyuchun1963@aliyun.com

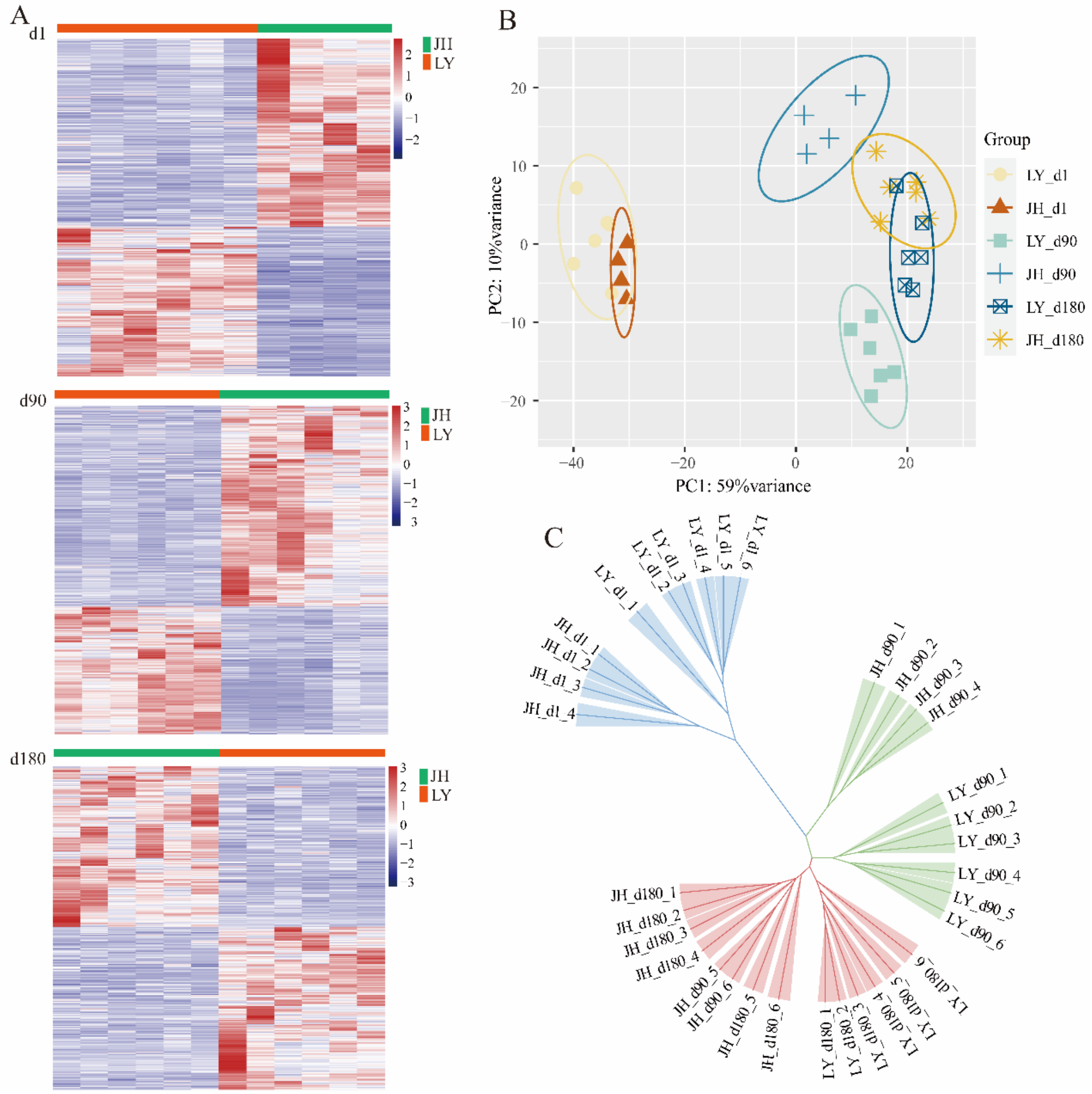

Figure S1. The cluster analysis of all samples, including JH and LY pigs at three different age stages (d1, d90 and d180). (A) The heatmap of all samples. (B) PCA analysis of all samples. (C) The circular hierarchical clustering of all samples.

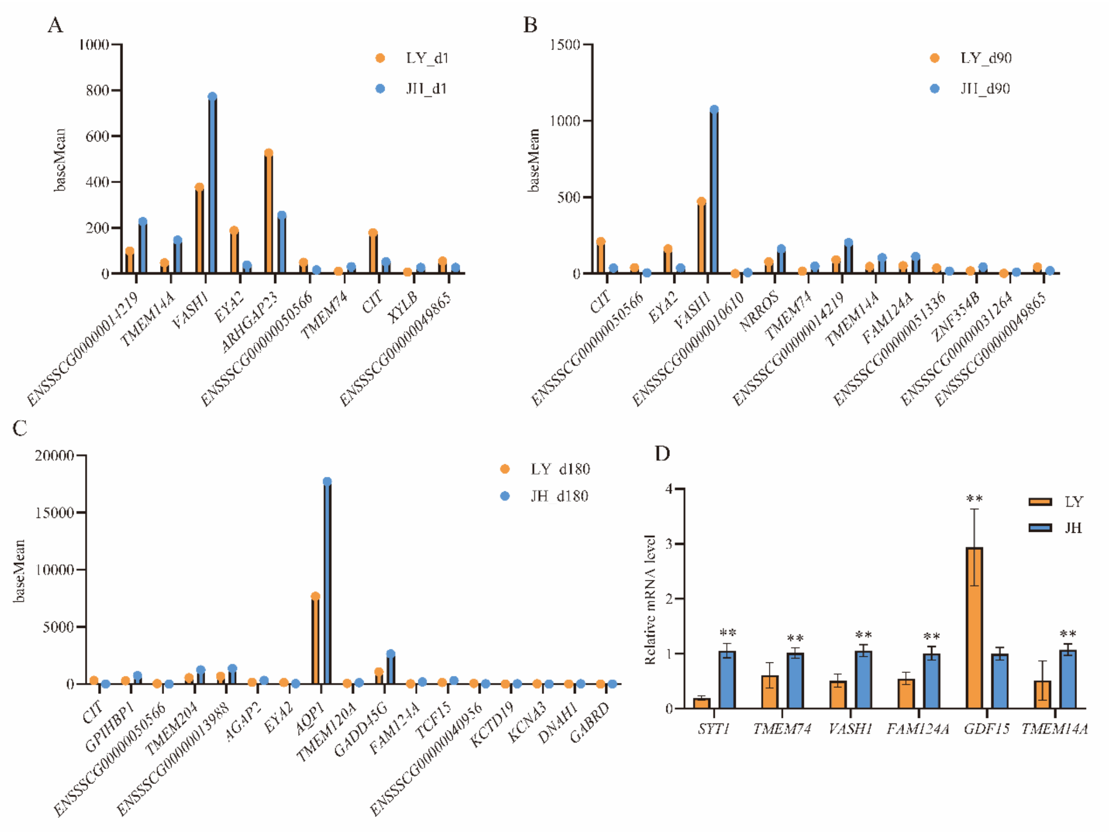

Figure S2. The expression level of DEGs between JH and LY pigs. (A-C) The baseMean expression level of DEGs calculated through "DESeq2" R package in d1, d90 and d180 groups, respectively. (D) The qPCR of gene expression levels between JH and LY pigs.

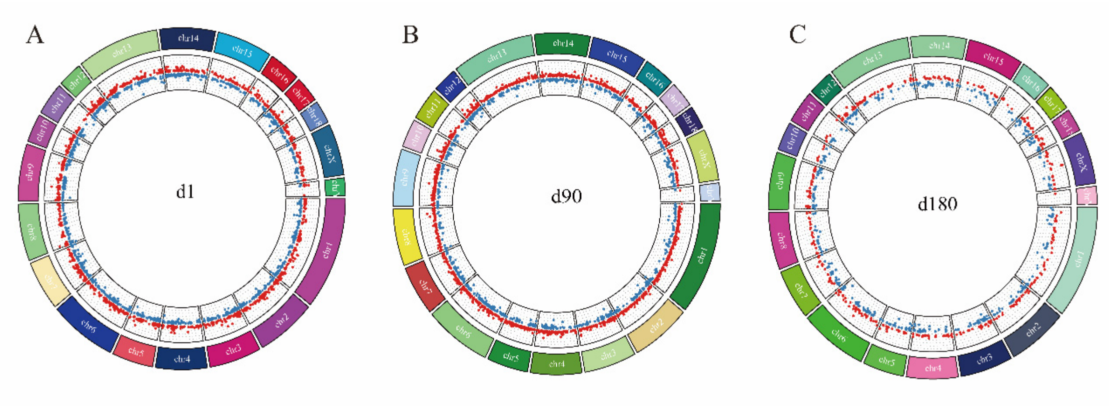

Figure S3. The distribution of DEGs across chromosomes. (A-C) The circular maps of density distribution of DEGs between JH and LY pigs in d1, d90 and d180 groups, respectively. The outermost color block means chromosomes, red dots mean up-DEGs, and blue dots mean down-DEGs.

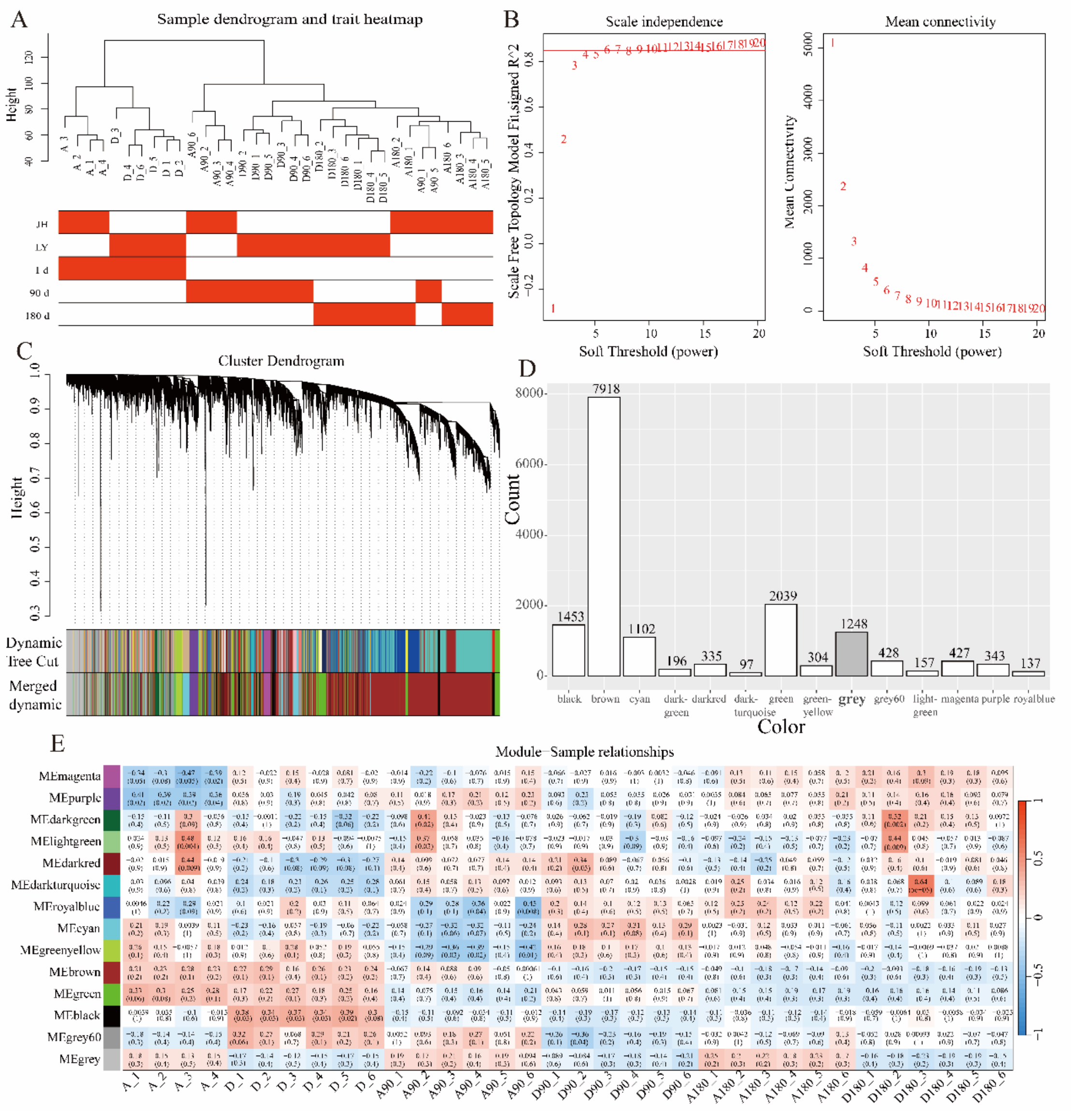

Figure S4. WGCNA of transcriptome. (A) Sample clustering plot. (B) Analysis of free-scale network topology for different soft-thresholding powers. (C) Division of gene modules. The branches of the same color were divided into the same gene modules. (D) Bar plot showing the number of gene in each module. (E) Heatmap showing the relationship between module and each sample. Red represents positive correlation, blue represents negative correlation. A represents 1-day-old JH pigs, A90 represents 90-day-old JH pigs, A180 represents 1-day-old JH pigs, D represents 1-day-old LY pigs, D90 represents 90-day-old LY pigs, D180 represents 180-day-old LY pigs.

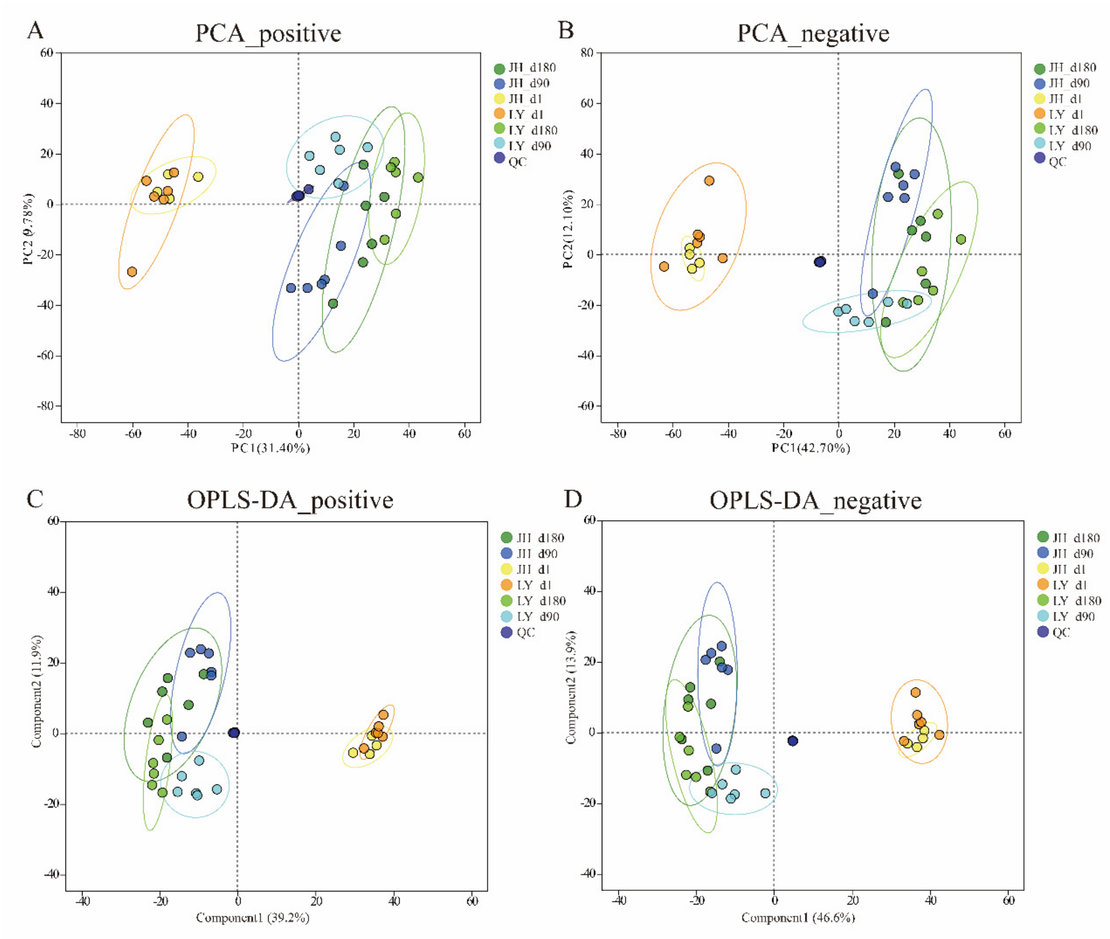

Figure S5. The cluster analysis of all samples. (A) PCA of all samples under positive ion mode. (B) PCA of all samples under negative ion mode. (C) OPLS-DA of all samples under positive ion mode. (D) OPLS-DA of all samples under negative ion mode.

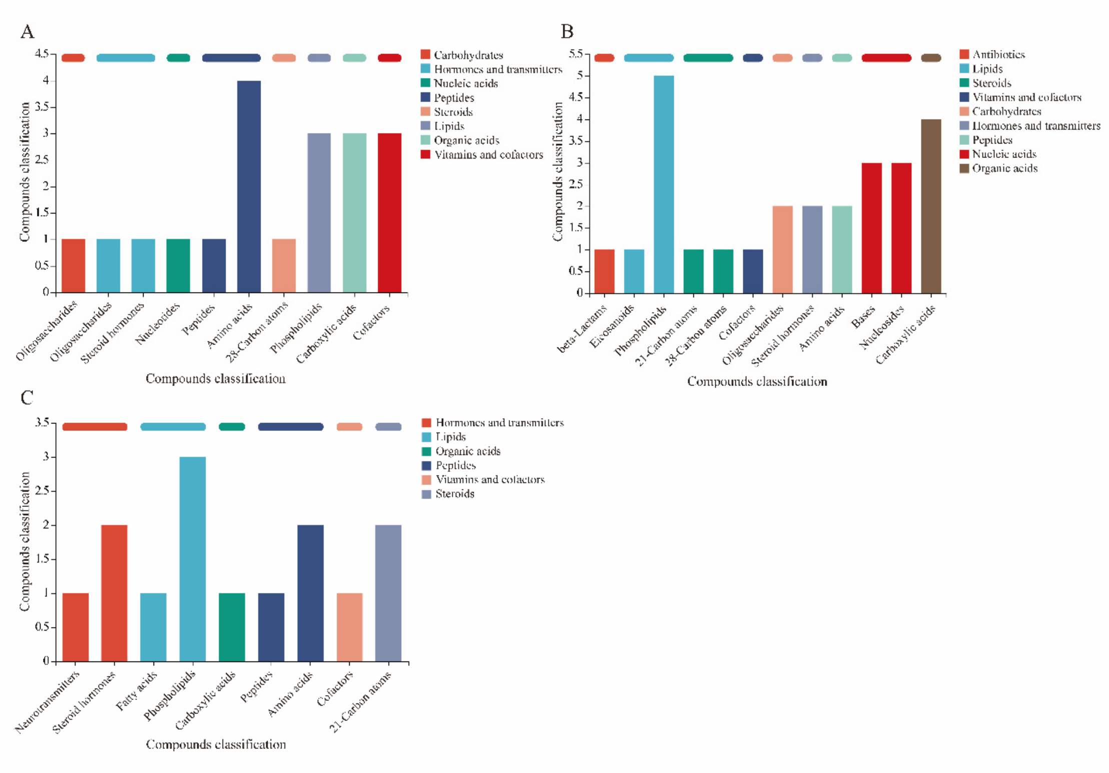

Figure S6. The classification of metabolites. (A) The classification of metabolites between JH and LY pigs in d1 group. (B) The classification of metabolites between JH and LY pigs in d90 group. (C) The classification of metabolites between JH and LY pigs in d180 group.

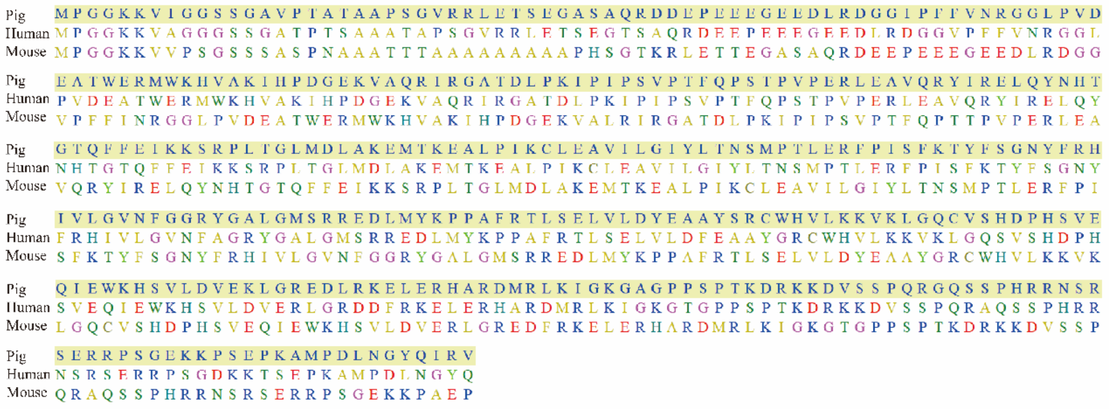

Figure S7. The homologous identity analysis of *VASH1* gene among pig, human and mouse.

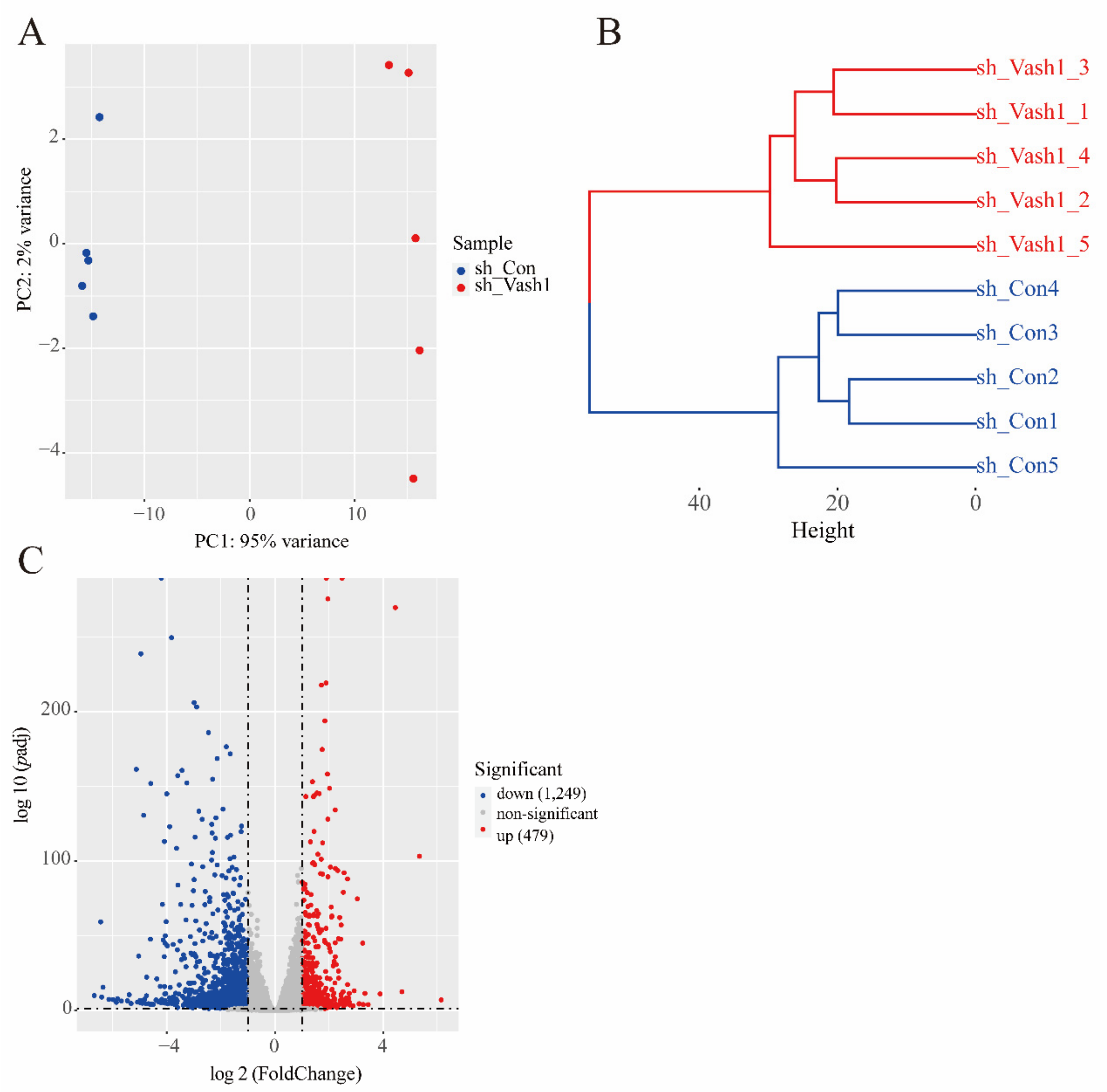

Figure S8. Transcriptomic analysis of Vash1-knockdown cells. (A) PCA analysis of all the samples. (B) NJ-tree cluster analysis of all the samples. (C) The volcano plots of genes between sh-Con and sh-Vash1 groups. Red dots mean up-DEGs, blue dots mean down-DEGs, and grey dots mean non-significant.
